# Fluorescently labeled *Pseudomonas syringae* DC3000 and 1449b wild-type strains constitutively expressing either eGFP, eCFP, or dsRED

**DOI:** 10.17912/micropub.biology.000595

**Published:** 2022-07-06

**Authors:** Jose S Rufián, Javier Ruiz-Albert, Carmen R Beuzón

**Affiliations:** 1 Departamento Biología Celular, Genética y Fisiología, Instituto de Hortofruticultura Subtropical y Mediterránea, Universidad de Málaga-Consejo Superior de Investigaciones Científicas (IHSM-UMA-CSIC)

## Abstract

Here we describe the generation of fluorescently labeled derivatives of the plant pathogen *Pseudomonas syringae* DC3000 and 1449b strains, with each derivative constitutively expressing either the enhanced green (eGFP), enhanced cyan (eCFP), or *Discosoma* sp. red (dsRED) fluorescent proteins. The fluorophore-expressing cassetes are stably located in a neutral locus in the chromosome, and its expression does not affect bacterial fitness, while allowing efficient detection by microscopy or flow cytometry. We have generated these strains as a complementary set of labeled strains to those previously generated in our laboratory, thus extending the range of applications.

**Figure 1. Validation of fluorescently labeled DC3000 and 1449b strains in regard to constitutive fluorescence emission and potential impact of constitutive fluorophore expression on bacterial fitness.**
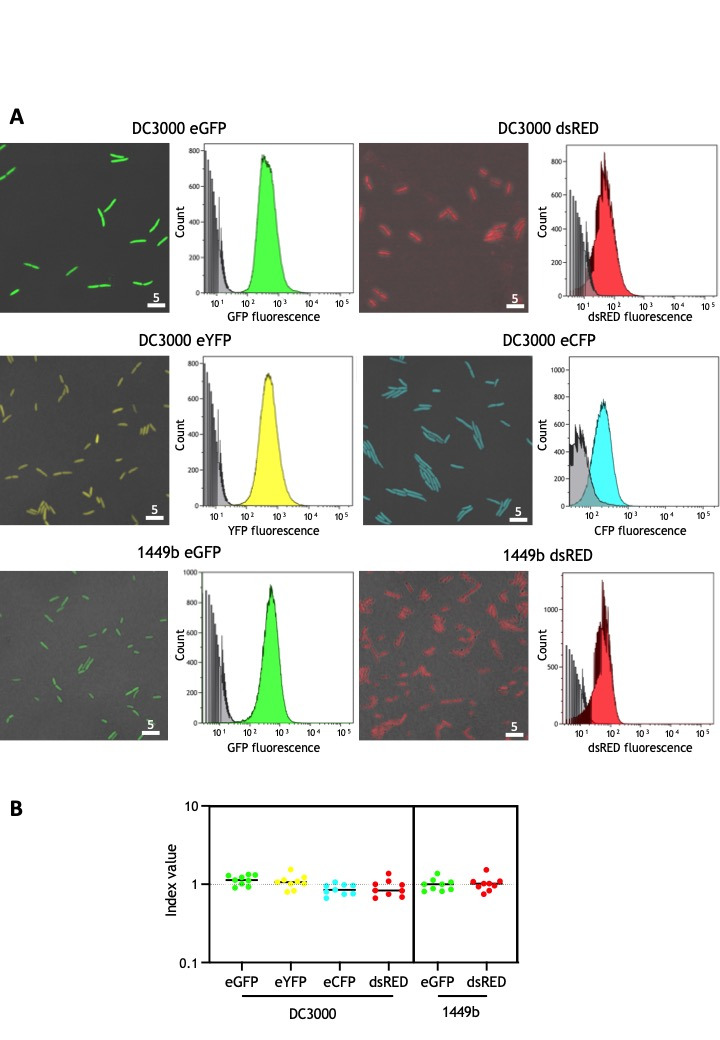
( **A** ) Confocal microscopy (left) and flow cytometry analysis (right) of the indicated bacteria grown in LB. Microscopy images are merged with the bright field to show that all bacteria fluoresce. Scale bar: 5 µm. For flow cytometry analysis, non-fluorescent bacteria were included as a control (in grey) ( **B** ) Competitive growth assays of bacteria grown in rich medium. Competitive index (CI) is defined as the mutant to wild-type ratio in the output sample divided by the same ratio in the input. None of the CI values were significantly different from 1.0 as established by Student´s *t* -test ( *P* <0.05) indicating that there was no significant growth difference between the labeled strains and the corresponding wild-type strains. Each CI value corresponds to the mean of nine replicates. Error bars correspond to the standard error.

## Description

In this work we describe the generation of fluorescently labeled derivatives of two model wild-type strains of the plant bacterial pathogen *Pseudomonas syringae:* (i) strain DC3000 belonging to pathovar (pv) tomato and able to colonize and multiply in Arabidopsis and tomato plants, and (ii) strain 1449b pv. phaseolicola, an effective pathogen of bean plants. Each fluorescently labeled strain expresses constitutively either the enhanced green (eGFP), enhanced cyan (eCFP), or *Discosoma* sp. red (dsRED) fluorescent proteins ( **Table 1** ). The fluorophore-expressing cassetes are stably located in a neutral locus in the chromosome, and its expression does not affect bacterial fitness, while allowing efficient detection by microscopy or flow cytometry. We have generated these as a complementary set of labeled strains to those previously generated in our laboratory (Rufián et al. 2016; Rufián et al. 2018; Rufián et al. 2021), thus extending the range of applications.

We have followed the same experimental procedures previously used for the generation of the DC3000 wild-type strain expressing the enhanced yellow (eYFP) fluorescent protein (Rufián et al. 2021), and of equivalent eGFP, eCFP, eYFP or dsRED-labeled strains of the bean pathogen *P. syringae* pv. phaseolicola 1448A and 1449B (Rufián et al. 2016; Rufián et al. 2018), as well as a number of mutant variants (Rufián et al. 2018; Rufián et al. 2021). The procedure is based on the use of a plasmid‑borne Tn7 delivery system that is introduced into *P. syringae* by tetraparental mating, and allows for the integration of a single copy of the corresponding fluorophore-expressing cassette in an intergenic region downstream the GlmS coding-gene (Lambertsen et al., 2004).

Constitutive fluorescence emission of all labeled strains was validated by confocal microscopy *,* with each bacterium emitting the corresponding fluorescence at a similar level ( **Fig 1A** ), and expression was confirmed to be homogeneous using flow cytometry on DC3000 and 1449b *in vitro* cultures, as previously described (Rufián et al. 2018) ( **Fig 1A** ). We also analysed any potential impact of constitutive fluorophore expression on bacterial fitness using competitive growth assays in rich medium, as previously described (Macho et al. 2007; Macho et al., 2016) ( **Fig 1B** ). We include the DC3000 strain constitutively expressing enhanced yellow (eYFP) protein, previously generated in our laboratory (Rufián et al. 2021), for comparison purposes ( **Fig. 1** ).

These fluorescently labeled set of strains will be valuable tools to follow *P. syringae* colonization of the plant apoplast via inoculation of individual strains in wild-type plants or plant mutant backgrounds, to analyse the relationships established within the plant between co-inoculated strains expressing different fluorophores, or to analyse bacterial infections of plants expressing fluorescently-tagged proteins or other cellular markers.

## Methods

**Bacterial strains and growth conditions**

Bacterial strains used in this work are listed in **Table 1** . *Escherichia coli* strains were grown at 37 ºC, while *Pseudomonas syringae* pv. tomato DC3000 (Cuppels 1986) and *Pseudomonas syringae* pv. phaseolicola 1449b (Teverson, 1991) strains were grown at 28 ºC, in all cases with aeration in Lysogeny Broth (LB) medium (Bertani, 1951). Antibiotics were used when appropriate at the following concentrations: ampicillin (Amp), 100 µg/mL for *E. coli* auxiliary 1 strain; gentamycin (Gm), 10 µg/mL for *P. syringae* labeled strains and 50 µg/mL for *E. coli* donor strains; chloramphenicol (Cm) 6 µg/mL for *E. coli* auxiliary 2 strain; nitrofurantoin (Nf), 50 µg/mL for *P. syringae* strains.

**Generation of bacterial strains**

For all strains, genes coding for the different fluorescent proteins, under the transcriptional control of the constitutive *E. coli* lac-promoter derivative PA1/04/03, were introduced into the chromosome of strain DC3000 using a plasmid-borne Tn7 delivery system (Lambertsen et al., 2004). Plasmids carrying the delivery system were introduced into *P. syringae* by tetraparental mating, as described by Lambertsen et al. (2004). In brief, cultures of *P. syringae* DC3000 as recipient strain (R), and *E. coli* strains acting as donor (D, strain DH5a carrying pBK-mini-Tn7(Gm)P _A1/04/03_ - *ecfp* / *eyfp* / *egfp* / *dsRed* ), auxiliary 1 (A1, strain SM10 *λ* pir carrying pUX-BF13), and auxiliary 2 (A2, strain HB101 carrying pRK600), were grown overnight in LB medium with aeration at 28ºC ( *Pseudomonas* ) or 37ºC ( *E. coli* ). LB was supplemented with 50 µg/mL Gm for donor strain cultures, 100 µg/mL Amp for A1, and 6 µg/mL Cm for A2. Mixtures for tetraparental mating were prepared with the proportion 3 (R): 1 (D): 1 (A1): 1 (A2), by measuring the OD600 of each overnight culture and adjusting by dilution as required. For each mixture, bacterial cultures with an OD600 of 4 were concentrated by centrifugation at 5000 rpm for 5 min, using 4 mL for the recipient strain and 1 mL for each of the *E. coli* strains. Two negative control mixtures were also prepared, one using only *P. syringae* and the other using all *E. coli* strains, keeping the aforesaid proportions. Each tetraparental mixture, with a final volume of 100 µL, was placed on top of a 0.22 μm millipore filter sitting on a well-dried LB plate, and incubated overnight at 28ºC. Filters carrying the mating samples were then collected with sterile tweezers into 1 mL of 0.9% NaCl, the bacterial mixture was resuspended by vortexing, concentrated by centrifugation at 10000 rpm for 2 min, resuspended into 100 µL of 0.9% NaCl, plated onto selective media (LB supplemented with 10 µg/mL Gm and 50 µg/mL Nf), and incubated at 28ºC for 2-3 days. All fluorescently labeled strains generated, together with the plasmids used for this purpose, are listed in **Table 1** . The single insertion of each expression cassette in the expected site of the bacterial genome, downstream the GlmS-coding gene, was validated by polymerase chain reaction (PCR) analysis with primers Tn7-GlmS and Tn7R109 (Lambertsen et al., 2004), and further confirmed by Southern blot analysis using *aacC1* (Gm ^R^ ) as a probe.

**Competitive bacterial growth assays**

The *in vitro* competitive index (CI) assays were carried out in LB medium as described previously (Macho et al., 2007; Macho et al., 2016). Competitive index assays allow for the direct comparison between the respective growths of co-inoculated strains within the same culture. In brief, for each *in vitro* assay, 500 mL of a 5 x 10 ^4^ cfu/mL mixed inoculum, containing equal numbers of cfu (colony forming units) of the wild-type strain and the corresponding derivative strain, was inoculated into 4.5 mL of LB medium and grown for 24 h at 28 ºC with aeration. Bacterial enumeration was then performed by serial dilution and sample plating onto LB agar, with and without 10 mg/mL Gm, to determine the precise ratio between co-inoculated strains. All colonies grown on LB plates supplemented with Gm displayed fluorescence corresponding to the expressed fluorophore.

**Microscopy**

Constitutive fluorescence emission of all labeled strains was validated *in vitro* by confocal microscopy, as previously described (Rufián et al. 2016). Images were taken using the Leica Stellaris confocal microscope (Leica Microsystems) and the following settings (excitation/emission in nm): eGFP (488/500 to 550), eYFP (514/525 to 575), eCFP (440/450 to 500), dsRED (558/570 to 630).

**Flow cytometry**

Fluorophore expression analysis by flow cytometry was performed on DC3000 and 1449b *in vitro* cultures for all labeled strains, in comparison with a non-fluorescent bacterial population used as a negative control, as previously described for GFP fluorescence (Rufián et al. 2016). Overnight cultures were washed and resuspended in 10 mM MgCl2 and analysed using a BD FACS Aria cytometer (BS Biosciences).

**Table:** 

**Strain**	**Relevant features**	**Reference**
DH5a	*Escherichia coli F-endA1 hsdR17 supE44 thi-1 recA1 gyrA96 relA1 ΔlacU189 f80 Δ-lacZDM15*	Hanahan (1983)
SM10 *λ* pir	*Escherichia coli thi thr leu tonA lacY supE recA::RP4-2-Tc::Mu* Km ^R^ *λpir*	Simon et al. (1983)
HB101	*Escherichia coli* K-12/B hybrid; Sm ^R^ *recA thi pro leu hsdRM* ^+^	Boyer and Roulland-Dussoix (1969)
DC3000	*Pseudomonas syringae* pv. tomato wild-type strain, Nf ^R^	Cuppels (1986)
1449b	*Pseudomonas syringae* pv. phaseolicola wild-type strain, Nf ^R^	Teverson (1991)
JRP21	DC3000 Tn7-eGFP, Gm ^R^	This work
JRP22	DC3000 Tn7-eYFP, Gm ^R^	Rufián et al (2021)
JRP23	DC3000 Tn7-eCFP, Gm ^R^	This work
JRP24	DC3000 Tn7-dsRED, Gm ^R^	This work
JRP14	1449b Tn7-eGFP, Gm ^R^	This work
JRP16	1449b Tn7- dsRED, Gm ^R^	This work
**Plasmid**	**Description**	**Reference**
pUXBF13	Helper plasmid expressing Tn7 transposase, Amp ^R^	Bao et al. (1991)
RK600	Conjugation helper plasmid, Cm ^R^ ColE1 oriV RP4 oriT	Kessler et al. (1992)
pBK-miniTn7(Gm) _PA1/04/03_ -ecfp-a	eCFP, Amp ^R^ , Gm ^R^	Lambertsen et al. (2004)
pBK-miniTn7(Gm) _PA1/04/03_ -eyfp-a	eYFP, Amp ^R^ , Gm ^R^	Lambertsen et al. (2004)
pBK-miniTn7(Gm) _PA1/04/03_ -gfpAGA-a	eGFP, Amp ^R^ , Gm ^R^	Lambertsen et al. (2004)
pBK-miniTn7(Gm) _PA1/04/03_ -DsRedExpress-a	dsRED, Amp ^R^ , Gm ^R^	Lambertsen et al. (2004)
		
